# Exploring mechanisms of diet-colon cancer associations through candidate molecular interaction networks

**DOI:** 10.1186/1471-2164-15-380

**Published:** 2014-05-17

**Authors:** David Westergaard, Jun Li, Kasper Jensen, Irene Kouskoumvekaki, Gianni Panagiotou

**Affiliations:** School of Biological Sciences, The University of Hong Kong, Pokfulam Road, Hong Kong, China; Center for Biological Sequence Analysis, Department of Systems Biology, Technical University of Denmark, Kemitorvet, Building 208, Lyngby, DK-2800 Denmark

## Abstract

**Background:**

Epidemiological studies in the recent years have investigated the relationship between dietary habits and disease risk demonstrating that diet has a direct effect on public health. Especially plant-based diets -fruits, vegetables and herbs- are known as a source of molecules with pharmacological properties for treatment of several malignancies. Unquestionably, for developing specific intervention strategies to reduce cancer risk there is a need for a more extensive and holistic examination of the dietary components for exploring the mechanisms of action and understanding the nutrient-nutrient interactions. Here, we used colon cancer as a proof-of-concept for understanding key regulatory sites of diet on the disease pathway.

**Results:**

We started from a unique vantage point by having a database of 158 plants positively associated to colon cancer reduction and their molecular composition (~3,500 unique compounds). We generated a comprehensive picture of the interaction profile of these edible and non-edible plants with a predefined candidate colon cancer target space consisting of ~1,900 proteins. This knowledge allowed us to study systematically the key components in colon cancer that are targeted synergistically by phytochemicals and identify statistically significant and highly correlated protein networks that could be perturbed by dietary habits.

**Conclusion:**

We propose here a framework for interrogating the critical targets in colon cancer processes and identifying plant-based dietary interventions as important modifiers using a systems chemical biology approach. Our methodology for better delineating prevention of colon cancer by nutritional interventions relies heavily on the availability of information about the small molecule constituents of our diet and it can be expanded to any other disease class that previous evidence has linked to lifestyle.

## Background

Nutrition is the cornerstone of an individual’s lifestyle, thus, understanding how diet influences metabolic regulation and how dietary interventions can improve health is a key scientific goal. At the same time, diet has a major influence on the overall quality of life beyond the prevention of diseases. Thus, even though the personalized approach to diet is the logical transition – much like the transition from pharmacology to personalized medicine – this task is extraordinary complex [1, 2]. Most foods are composed of hundreds of bioactive compounds, often interacting synergistically, with varied concentrations and several biological targets with different affinities and specificities. However, since nutritional trials are not designed as mechanism-based preclinical studies little is known about these molecular targets. Observational studies performed on populations with distinct diet preferences, often coupled with sophisticated statistical analyses, have offered some associations between certain diseases and foods [3–5]. Even though in some cases, these kind of studies have led to further mechanistic investigations that resulted in elucidation of the bioactive natural compounds, they suffer in two ways: (*a*) they are phenomenological in nature, (*b*) they are very restricted in the confined disease phenotype space that is under study.

On the other hand, numerous successful stories of FDA-approved therapeutic agents derived from phytochemicals: *Hycamtin* (GlaxoSmithKline), *Navelbine* (Pierre Fabre Pharmaceuticals Inc.), *Taxol* (Bristol-Myers Squibb), *Taxotere* (Sanofi-aventis) among others, have propelled the research on how dietary bioactive compounds interact with target proteins and perturb key signaling pathways. Thus, the second approach that we often encounter in nutritional studies is the *in vivo* or *in vitro* investigation of the beneficial effects of common phytochemicals, such as specific polyphenols that are present in a variety of fruits, vegetables, and other dietary botanicals. An abundant literature has shown that polyphenols can, among others, trigger apoptosis in cancer cells through the modulation of a number of key elements in cellular signal transduction pathways linked to apoptosis (caspases, *bcl-2* genes) [6] and modulate epigenetic alterations in cancer cells [7]. However, in such studies the major limitation is that the possible therapeutic value of the phenolic compound of interest is evaluated while ignoring the chemical background of the diet, which is probably one of the reasons for contradictory results from different research groups on the same compounds. Therefore, there is a clear need for more systematic studies to identify those dietary factors that influence the most, reveal their synergistic interactions, and uncover the mechanisms of action.

Cancer research in the last 20 years has brought to the fore a dramatic amount of information at the molecular level, leading to an overwhelming number of possible pharmaceutical targets for drug discovery. However, the redundancy and interconnection of the many regulatory pathways that are involved in cell replication, growth and apoptosis, as well as the capacity of mutations in cancer cells, is a significant barrier for drug development using targeted approaches. These hard limitations encountered with the targeted approach are contributing to prompt us to reconsider the global fight against cancer, especially for cancers that are proven to be intrinsically or partly related to lifestyle factors, such as diet. Beyond the specific aspect of cancer prevention, understanding the capacity of the body to maintain health homeostasis is a genuine subject of study for which a methodological approach needs to be considered. Taken separately each regulatory cascade interaction may not help framing an operational understanding of health homeostasis whereas a more global view, where the concomitant activity of the largest number of targets with respect to the wave of external agent exposure, such as dietary molecules, could be scrutinized as a complex interaction network. There is a general consensus that in the new era of nutritional research, systems analysis of normal and nutrient-perturbed signaling networks is required for identifying critical network nodes targeted through nutritional intervention of either preventive or therapeutic fashion [8]. Borrowing chemoinformatics methods, well established and widely used in drug discovery research, could help us understand the complex interaction network between dietary small molecules and biological systems. In line with this, we present here a systems chemical biology approach that provides a fundamental foundation for understanding which processes involved in the onset, incidence, progression and severity of colon cancer are modulated by dietary components. We selected colon cancer as a case study not only because it is one of the most aggressive cancers and the fourth most commonly diagnosed, but also because colon cancer seems not to be a consequence of aging but of eating behavior [9, 10]. Nevertheless, the methodology proposed here is applicable to any large-scale diet-disease association study, where information about the small molecule constituents of the diet is available.

## Results

### The chemical space of diet associated with colon cancer

**Figure 1:**
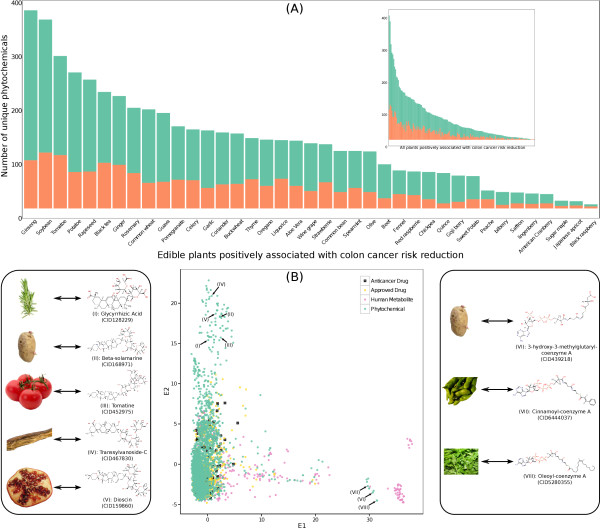
**The chemical space of plants associated with colon cancer. (A)** A sub list containing 39 plants that are considered typical for western diet; the embedded figure shows the compound distribution as found in the 159 edible and non-edible plants. Green indicates total number of compounds, while orange indicates compounds that were found as exact match in CHEMBL; **(B)** Clustering of the plant compounds (green), human colon metabolites (yellow), FDA approved drugs (purple) and anticancer FDA approved drugs (black cross) based on 1027 chemical descriptors. Selected compounds ((i)-(viii)) from the plant chemical space that show either no chemical similarity with the other groups of compounds are shown on the left or low chemical similarity only with drugs are shown on the right.

Using an *in-house* database developed by Jensen *et al.,* (2014) [11] through text mining of 21 million abstracts present in PubMed, we investigated here the role of dietary small molecules present in plants (edible and non-edible) with an established phenotype against colon cancer. As shown in Figure 1A, we have identified 158 plants that have been positively associated in the literature with colon cancer, with 39 of them being part of a common diet, e.g. celery, garlic, thyme, among others (Figure 1A). From our *in-house* database we could also retrieve molecular information for these plants for 3,526 unique phytochemicals. It is quite interesting that despite the fact that all these plants have been positively associated with colon cancer reduction, the majority of phytochemicals (2,023) are plant specific (found only in one plant). The number of compounds associated with each plant varied considerably (Figure 1A), with a median value of 41 compounds per plant and reaching as high as 392 compounds for ginseng. Not surprisingly, as shown in Figure 1A, common foods have been studied more thoroughly than other plants and have a median value of 129 compounds per food. Nevertheless, a group of phytochemicals has been detected in a very large number of plants associated with colon cancer; quercetin (N = 51), gallic acid (N = 43), vitamin P (N = 43), gamma-sitosterol (N = 42) and kaempferol-3-O-rutinoside (N = 38). Since our objective was to obtain a mechanistic understanding of the association between plants and colon cancer we examined how many of the 3,526 phytochemicals are present as an exact match in ChEMBL database, one of the largest repositories of chemical-protein interaction data. As shown in Figure 1A, roughly for one third of the compounds present in each plant there are available experimental data for their interactions with the human protein space. We could find, in total, an exact match in ChEMBL for 1,663 phytochemicals, while 887 have a chemical similarity (TC > 0.85 & a difference in molecular weight less than 50 g/mol) with at least one other chemical in ChEMBL. For the remaining 976 compounds no information is available for possible protein targets.

The chemical space of the phytochemicals from plants associated with colon cancer was evaluated using 1,027 chemical descriptors. To obtain a holistic view of this chemical space we compared it with FDA approved drugs and the human metabolites involved in the colon metabolic network developed by Agren *et al.,* (2012) [12]. Interestingly, a large percentage of the plant phytochemicals shows a high degree of chemical similarity with metabolites of the human colon metabolic network pointing out that the therapeutic effect of these plants could be mediated at a metabolic level (Figure 1B); however, we should not overlook the high similarity between FDA drugs and plant phytochemicals and especially anticancer drugs (Figure 1B). On the other hand a large number of phytochemicals (left upper corner of Figure 1B) has a very unique chemical profile with no similarities to either the drug space or the colon metabolic network. Examples of such compounds (Figure 1B) are glycyrrhizic acid (in rosemary), beta-solamarine (in potato), tomatine (in tomato), transsylvanoside C (in liquorice) and diocine (in pomegranate). The above compounds are present in just a handful of edible and non-edible plants that have been associated to colon cancer. In the lower right part of Figure 1B we find compounds with structural similarities solely with approved drugs, e.g. 3-hydroxy-3-methylglutaryl-coenzyme A (in potato), cinnamoyl-coenzyme A (in soybean) and oleoyl-coenzyme A (in coriander). The source of these compounds is again restricted to a few edible and non-edible plants.

### An interactome map of candidate colon cancer targets and diet

**Figure 2:**
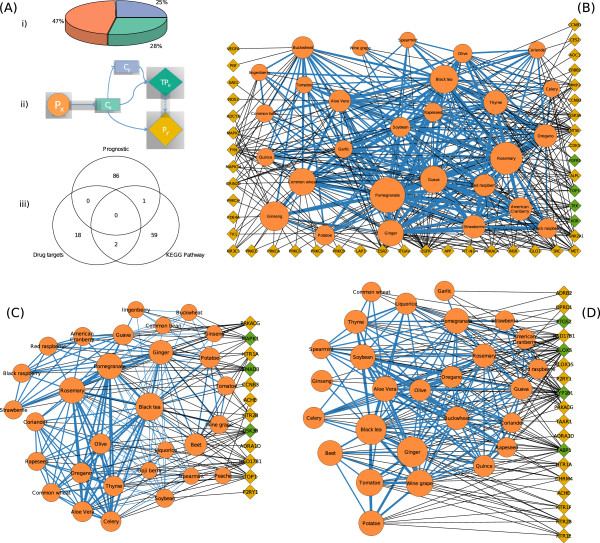
**The interactome space of the edible plants associated to colon cancer. (A)** (i) The pie chart offers information for the compounds present in these edible plants; phytochemicals found as exact match in ChEMBL (orange), phytochemicals having at least one similar small molecule present in ChEMBL (blue) phytochemicals that do not fulfill any of the above criteria (green); (ii) A graphical representation of how the interactome map between edible plants and candidate colon cancer protein targets was generated: (a) an edible plant (orange circle; Px) contains a phytochemical (box; Cx) which interacts directly with a target protein (green diamond; TPx) or through a first-degree neighbor (yellow diamond; Py) of TPx, (b) the phytochemical Cx is structurally similar with a compound (Cy) that interacts with the target protein (TPx). Straight lines represent verified interactions while dashed lines represent predictions; (iii) The Venn diagram shows the overlap between the proteins in the three candidate colon cancer target sets; **(B)** A plant-protein interaction network based on the interactions between phytochemicals, FDA approved colon cancer drug targets and their first-degree neighbors. The size of the plant node is proportional to the number of proteins that its molecular components target. The width of the edge connecting two plants reflects how many protein targets the plants share. **(C)** A plant-protein interaction network using as a candidate target space the KEGG colon cancer pathway. **(D)** A plant-protein interaction network using as a candidate target space the colon cancer prognostic gene signatures (Oh *et al.,* 2012) [13]. The color of the nodes in **(B)-(D)** is according to (Aii).

To unravel the interactions associated with diet and colon cancer we studied the complex interaction networks of the small molecules present in the 158 plants and the candidate colon cancer target space. By “candidate” here we mean proteins that are potentially involved in the onset and development of colon cancer and fall in one of the following categories (Figure 2A), (i) proteins that are established targets of the FDA approved colon cancer drugs (N = 20) and their first degree neighbors (N = 1,224); ii) proteins that participate in the KEGG colon cancer pathway (N = 62) and their first degree neighbors (N = 1,588); iii) proteins characterized by Oh *et al.,* (2012) [13] as colon cancer prognostic signature (N = 87) and their first degree neighbors (N = 870). First-degree protein neighbors were included since cancer-related proteins are more likely to act as hubs in protein interaction networks. This feature of cancer proteins makes them amenable to activity disturbances through ligand binding to their protein neighbors. To ensure the biological validity of the interactions in the context of colon cancer only proteins for which there is positive evidence of expression in the colon according to the Human Protein Atlas [14] were included. As shown by the Venn diagram in Figure 2A there is very low overlap between the three categories (not taking into account the first-degree neighbours). The total number of unique proteins is 181 (and 1,708 unique first degree neighbors). For the association of the plants with the candidate colon cancer protein space through their molecular components we considered direct (a) and indirect (b) interactions: (a) a plant contains a compound that was found as an exact match in ChEMBL to interact with either the set of 181 or 1708 proteins; (b) a plant contains a compound structurally similar to a compound in ChEMBL that interacts with the set of 181 proteins. Only high confidence interactions, either chemical-protein or protein-protein, were kept (see Methods).

(i) In total, 105 plants (33 edible) were found to interact with 4 target proteins (TEK, KDR, FGR1 and TOP1) of the FDA approved colon cancer drugs and 43 of their first-degree neighbors. The mean number of proteins from this category targeted by each plant was 6 (9 if we restrict the analysis to the common edible). The most targeted proteins of the compounds from edible plants were EGFR (targeted by 26 edible plants or 79% of the total edible plants in our database), NT5E (24 edible plants), ESR2 (20 edible plants), CSNK2A1 (17 edible plants) and FYN (17 edible plants). None of the above is an FDA colon cancer drug target but all are first degree neighbors of the drug targets. Similar results were observed when looking into the most targeted proteins of the non-edible plants. The interaction network between common edible plants and proteins from this category is shown in Figure 2B. The FDA approved drugs used against the 4 colon cancer proteins that are targeted by small molecules present in the edible plants, are irinotecan (TOP1) and regorafenib (TEK, KDR, FGR1). Pomegranate (N_small molecules_ = 13, targeting N_proteins_ = 23), rosemary (N_small molecules_ = 13, targeting N_proteins_ = 20), black tea (N_small molecules_ = 12, targeting N_proteins_ = 16), ginseng (N_small molecules_ = 14, targeting N_proteins_ = 17) and wheat (N_small molecules_ = 10, targeting N_proteins_ = 11) are the common foods of our diet with small molecules targeting the most this protein space. The mean connectivity ratio for the edible plants of Figure 2B is 3.7 (calculated as the sum of all edge weights divided by the number of edge weights).

(ii) Similarly, we explored the interaction pattern of the phytochemicals in the 158 plants with the proteins in the KEGG colon cancer pathway and their first-degree neighbors. In total 12 proteins are targeted by 73 plants through 32 unique small molecules. From the common edible plants (Figure 2C) 11 were found to have compounds targeting 3 proteins (MARK1, SMAD3, GSK3B) of the KEGG colon cancer pathway and 28 targeting the remaining 9, which are first-degree neighbors of the proteins in the KEGG colon cancer pathway. Black tea (N_small molecules_ = 5, targeting N_proteins_ = 7), ginger (N_small molecules_ = 4, targeting N_proteins_ = 5), rosemary (N_small molecules_ = 5, targeting N_proteins_ = 6) and pomegranate (N_small molecules_ = 4, targeting N_proteins_ = 6) are the common foods of our diet with small molecules targeting the most this protein space. Interestingly, the pattern we observe in Figure 2B and C does not depend on the actual number of compounds present in each plant. Soybean, tomato, potato and guava, among others, are edible common plants with a large number of compounds however, none of them appears to target a large part of the candidate colon cancer protein space. The proteins from this category that are targeted the most from the edible common plants are HSD17B1 (43% of edible plants), TOP1 (37%), GSK3B (37%), SMAD3 (27%) and PRKACG (27%). GSK3B and SMAD3 are proteins involved in the KEGG colon cancer pathway and the remaining three are first-degree neighbors. The mean connectivity ratio of the network of Figure 2C is 1.7.

(iii) The last candidate colon cancer protein space under study was the prognostic signatures based on gene expression data [13]. From the 87 proteins and their 870 first-degree neighbors that this targeted space consists of, only 5 proteins designated as prognostic signatures and 17 of their first-degree neighbors are targeted by the chemical space of the plants. In this category we found most of the edible plants: 41 small molecules from 37 edible plants target 4 proteins that constitute colon cancer prognostic signatures and 14 that are their first-degree neighbors. The edible plants with the highest activity in this target space are black tea, ginger, tomato and grape, which interestingly share none of the active compounds. The most targeted proteins are CYP1B1, ALOX5 and FABP1 and the mean connectivity ratio of the network of Figure 2D is 3.9.

The candidate colon cancer protein space that is targeted by plants with an established phenotypic effect against colon cancer allowed us to get a better insight on the biological and network properties of these 79 proteins. By using the Database for Annotation, Visualization and Integrated Discovery (DAVID) [15], the 79 proteins were annotated according to the protein domains as found in the InterPro database [16], and subsequently tested for enrichment. Then these domains were clustered using the integrated functional clustering in DAVID (default settings). Only clusters in which at least one InterPro family had a p-value < 0.05 (corrected for multiple testing using the Benjamini-Hochberg approach) were considered significantly enriched. We found three enriched clusters, in which the cluster with the highest enrichment score consisted mostly of kinase domains. The other two domains are related to cell division (Cyclin domains) and growth (EGF receptor domain). We also computed the intra-cluster distance between two proteins as the average shortest path distance between all pairs in this set of 79 proteins and we found a value of 2.2 (with the longest to be 5). Even though we started with three discrete candidate colon cancer target sets, the topological coefficients reveal a strong communication between the proteins, which may offer an explanation why, despite the differences in the observed candidate target space of each plant, they all produce the same phenotypic effect.Seventy two (72) plants, of which 28 edible (celery, thyme, coriander, oregano, olive, ginger among others) were found to contain compounds that target proteins from all three candidate colon cancer protein space. 97 plants (35 edible) target proteins from two protein spaces and 117 plants (37 edible) target proteins from one. For 41 of the plants, of which 2 were common edible plants (chickpea and sugar maple), we found no compounds to interact with any of the four categories of the candidate colon cancer target space. The number of the chemical compounds associated to chickpea and sugar maple could not explain this observation since these two edible plants are not the least characterized (Figure 1B). On the other hand, the average number of compounds (14.6) assigned to the non-edible plants showing no interactions with the colon cancer targets was significantly lower than the rest of the plant space of our study.

### The “hot” colon cancer space

**Figure 3:**
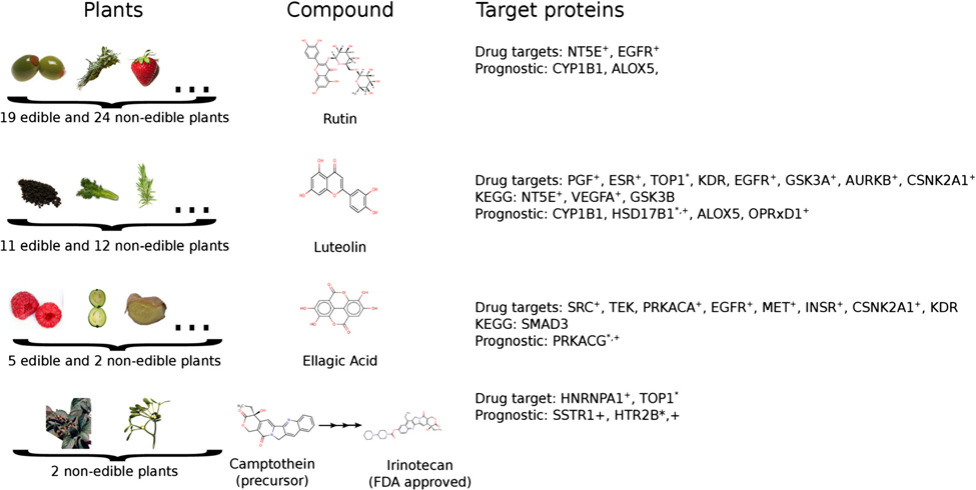
**Phytochemicals present in edible and non-edible plants with significant interest in connection to the candidate colon cancer target space.** *: Target also found in KEGG Pathway. +: Protein is a Px (see Figure 2).

The next step was to take a closer look on these phytochemicals and proteins that could hold the key for understanding the mechanism behind the positive phenotype of the particular edible and non-edible plants against colon cancer. In Figure 3 we present some of the chemical structures that caught our attention in this analysis. For example, rutin, is a compound present in 19 edible (olive, thyme, strawberry, among others) and 24 non-edible plants. Rutin targets two proteins, namely NT5E and EGFR, which both interact with established colon cancer drug targets and two proteins that are part of the colon cancer prognostic signature gene set (CYP1B1 and ALOX5). Another interesting compound is luteolin, a compound found in 11 edible (black tea, celery, rosemary, among others) and 12 non-edible plants. Luteolin has an interesting interaction network that includes 15 proteins from three candidate colon cancer target spaces, making it undoubtedly one of the most “hot” dietary compounds. Ellagic acid, present in 5 edible (strawberry, guava, ginger, rosemary and cranberry) and 2 non-edible plants targets as well proteins from all three candidate protein sets (Figure 3). Another interesting compound is camptothecin that was found in 2 non-edible plants. Camptothecin is actually a precursor of irinotecan, a drug primarily used for the treatment of colon cancer.

**Figure 4:**
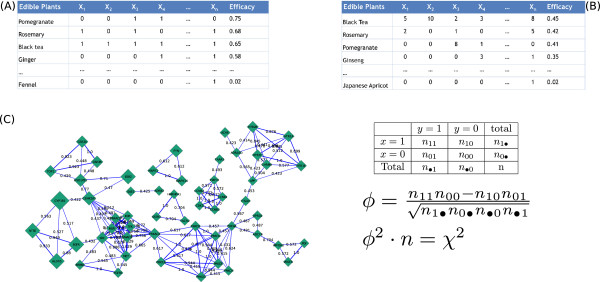
**The highly active edible plant space and the highly correlated colon cancer target space. (A)-(B)**: The two tables show the efficacy of each plant (for details see Methods) calculated using either **(A)**: a binary index; 0 = no phytochemicals in the plant interact with the protein (proteins in the tables are indicated as X_1_-X_n_), 1 = at least one phytochemical in the plant interacts with the protein, or **(B)**: the number of unique phytochemicals present in the plant that interact with the protein. **(C)**: A network of candidate colon cancer proteins connected by the phi correlation coefficient. Labels on the edges are the phi correlation coefficients. The network shows only significant correlations (p < 0.05, Bonferonni corrected). Correlation coefficients are calculated using the knowledge of plant-protein interactions, and thus a large correlation coefficient indicates that these two proteins are targeted by a common set of plants. The node size is proportional to the actual number of plants targeting a protein. The equations used to calculate the correlation coefficients are shown on the right, in which the squared phi coefficient is related to the Chi-squared test statistic by the number of samples.

Subsequently we calculated the efficacy *E(P)* of each plant as the ratio of the number of candidate colon cancer proteins targeted by plant *P* to the total number of candidate colon cancer proteins targeted by all plants (79 in total). As shown in Figure 4A, the highest efficacy was observed for pomegranate (59 targeted proteins, *E =* 0.75), rosemary (54 targeted proteins*, E =* 0.68), black tea (51 targeted proteins, *E =* 0.65) and ginger (46 targeted proteins, *E =* 0.58) from the edible plant space and *Gingo biloba* (*E =* 1), *Butea monosperma* (*E =* 0.48), *Withania somnifera* (*E =* 0.48) and *Galphimia glauca* (*E =* 0.43) from the non-edible plant space. If we take into consideration the actual number of compounds present in the plants that target each of these proteins the picture is slightly different (Figure 4B). The most promising edible plant interacting with the candidate colon cancer protein space now appears to be black tea with an efficacy of *E* = 0.45, while ginseng reaches an efficacy of *E =* 0.35. From the non-edible plants, *Ginkgo biloba* shows the highest weighted efficacy followed by *Withania somnifera* and *Butea monosperma*.

In the last part of the analysis we tried to develop the necessary statistical framework in order to achieve the main objective of our study; to actually reveal the protein space that may explain the observed anti-cancer phenotype of these edible and non-edible plants. Due to its complex chemical background, the way that diet induces particular phenotypes must be fundamentally different from the drug mode of action (one compound - one, or more target(s)). We calculated the correlation coefficients based on the plant-protein interaction patterns of Figure 4A. In Figure 4C we have only included the significant correlations (p < 0.05, Bonferonni corrected) that show some very interesting patterns in the candidate colon cancer target space. There are several small networks of proteins that are consistently targeted, or avoided by these plants. The significance of this analysis is not that it further reduces the candidate colon cancer target space to 55 proteins (Figure 4C) but mainly that it allows to formulate hypotheses on the sets of proteins that could be of significant interest as potential targets, either through dietary interventions or polypharmacology. The smallest networks consist of only three proteins (e.g. MAPK1/FYN/MAPK14, HNRNPA1/SSTR1/TACR2), whereas the largest one of 41 proteins. However, as shown in Figure 4C, this large network could be viewed as five sub-networks that are bridged via PDE4A (connecting three sub-networks) and PRKACA (connecting two sub-networks). As can also be seen in Figure 4C, there are a lot of edges with high correlation coefficients, but when taking the node size into account (the number of plants actually targeting a protein) the number of interesting clusters decreases. For instance, in the TOP1/GSK3A/GSK3B cluster all edges have correlation coefficients > 0.923 and a relatively large node size, indicating that these three proteins are targeted by a lot of plants as a group (indicated by the high correlation coefficient). Another cluster in Figure 4C with high correlation coefficients and a relatively large node size is the NT5E/ALOX5/EGFR. CYP1B1, despite being the largest node in this network, shows very poor correlation (max(phi_CYP1B1_) = 0.563). This is probably due to the fact that nearly all compounds in the drug development pipeline are screened against this target in ADME assays. In that sense this target is most likely not as interesting as the aforementioned protein clusters when it comes to explaining the observed colon cancer phenotypes of particular plants as the result of synergistic interactions of small molecules.

### Metabolic regulation by dietary components

Two of our previous observations, the fact that the majority of the plant phytochemicals appear structurally similar to the assigned metabolites in the human colon metabolic network (Figure 1B) and that 43 plants with a known phenotype against colon cancer have no compounds interacting with the candidate colon cancer protein space, was the motivation to study the possible metabolic regulation triggered by dietary components. The colon metabolic network consists of 2,934 metabolites and 1,773 enzymes involved in 3,060 reactions. In the 158 plants that have been positively associated with colon cancer reduction there are 122 phytochemicals that are an exact match to one human metabolite and 13 more that are structurally similar to 10 human metabolites. We make the assumption that these phytochemicals perturb a human metabolic reaction in the colon only if they appear in the enzymatic reaction as substrates. Based on this, we found in total 570 metabolic reactions in colon to be affected by plants. From the edible plant space, soybean, rapeseed, potato and ginseng are the ones with the highest influence in the colon metabolic regulation by perturbing 421, 225, 210 and 196 of metabolic reactions, respectively. If we look specifically at the 43 plants that are not linked with any of the candidate colon cancer protein space, we see that only chickpea contains phytochemicals that are involved in 76 metabolic reactions in the colon.

**Figure 5:**
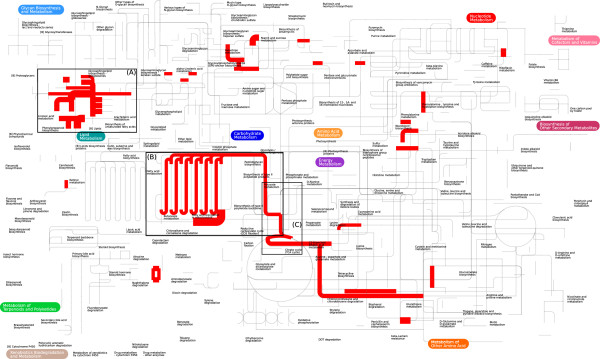
**The metabolic pathways of the human colon metabolic network (Agren** ***et al.,*** **2012) [**[12]**] influenced mostly by phytochemicals present in plants associated with colon cancer.** We highlighted the reactions in pathways that contain metabolites as substrates present in more than 20 plants. The width of the edge is proportional to the number of plants targeting a reaction in that pathway. We have zoomed in on lipid metabolism **(A)**, fatty acid **(B)**, pyruvate metabolism and TCA cycle **(C)**.

The observation that the regulation of the human metabolic network is under the control of signaling pathways often altered in cancer has shifted a lot of attention to cancer metabolism [17]. This has actually revealed the therapeutic potential of metabolic targets in cancer with important implications in the development of anticancer drugs. From Figure 5 we could actually get a visual representation of the metabolic processes in colon that are mostly targeted by the plants associated with colon cancer. Interestingly, the most targeted parts of the colon metabolic network are the lipid, fatty acid and pyruvate metabolism as well as the TCA cycle. Our findings are to a great extent in agreement with the analysis performed recently by Hu *et al.* (2013) [18], who used gene expression profiles gathered over the last decade to investigate the global shift in metabolic gene expression between and within cancers, including colon cancer. In their study, tumor-induced mRNA expression changes in lipid metabolism and fatty acid biosynthesis were associated with several cancer types. Even more interesting were the findings on colon cancer that were further validated by measurement of metabolite levels; there was observed a significant decrease in citrate concentration in tumor samples as well as a down-regulation of the pyruvate dehydrogenase complex that controls the majority of glucose carbon flux into the TCA cycle. Monitoring the levels of the TCA cycle intermediates in colon cancer patients after introducing specific dietary interventions could offer additional evidence for the mechanism that associates plants with colon cancer.

## Discussion

The term “exposome”, which is used to describe the totality of environmental exposures (e.g. diet, air pollutants, lifestyle factors) over the life course of an individual, has been proposed as a critical entity for disease etiology that complements genome information [19–21]. Diet is certainly one of the most dynamic expressions of the exposome and one of the most challenging to assess its effects in health homeostasis and disease development, due to its many components and their temporal variation. Recognizing, understanding and interpreting the interplay between diet and biological systems may contribute to the weight of evidence for assigning causality to a diet-disease association. Therefore, in order to open up new avenues to disease prevention through diet interventions it is crucial to provide insights into the mechanisms by which exposure to the chemical space of food might be exerting its effects.

Towards this direction we used colon cancer as a proof-of-concept for developing the necessary toolbox for a more cohesive view of diet exposure. From our systematic analysis of the candidate colon cancer target space, consisting of ~1,900 proteins, we identified a sub-set of 79 and further reduced it to 55 proteins that may reflect the mechanism by which the small molecule constituents synergistically define a food’s anti-cancer activity. This is in our opinion the most important contribution of our study; we go beyond the one compound-one target paradigm that has been extensively used in drug discovery and is often borrowed to explain the mode of action of dietary interventions. In contrast, here we identified statistically significant small protein clusters, from a pre-defined candidate colon cancer related space (avoiding in that way noise from uncurated protein interactions), that are targeted by dietary small molecules in a highly correlated manner. We have demonstrated that plants with different molecular profile can be associated to colon anticancer activity, as long as their protein targets are part of the same disease space.

Furthermore, we attempted to rank the efficacy of the plants associated to colon cancer using a simple scoring system. Taking again into consideration all the compounds present in each plant and their interaction profile with what we called “the hot” protein colon cancer space (consisting of 79 proteins) we found black tea, rosemary, pomegranate and ginseng leading the list of edible plants. It would certainly be very interesting to perform a comparative study, using a model animal system for colon cancer with edible plants that are ranked high and low in our list and verify in what degree these predictions stand true. Actually this list can be further expanded to any other edible or non-edible plant without known association to colon cancer as long as the chemical profile of the plant is adequately defined.

One of the major limitations in phenotypic screening studies is that it is practically impossible to test all foods against all disease phenotypes. However, analyses like the one performed here can lead to the identification of more foods with similar phenotypic effect based on the protein target space of their molecular components. Thus, our methodology for better delineating the prevention of human diseases by nutritional interventions relies heavily on knowing the small molecule constituents of our diet. While until recently this was a major obstacle to perform nutritional systems chemical biology studies, we have contributed significantly in this direction [11] by developing a state-of-the art database (currently *in-house* but soon part of it will become publicly available) with information on 16,102 plants, their small molecules constituents (20,654) and the human disease phenotypes (1,592) associated with these plants. This database offers a unique platform for performing global analysis of our diet-exposome for elucidating the synergistic interactions of the small molecules that yield specific phenotypes and their protein targets and hopefully will contribute in the future towards personalized nutrition based on the disease risk of the individual.

Last but not least, we should acknowledge the limitations of our study, mainly attributed to data incompleteness in relation to the phytochemical content of plants, their therapeutic effect on diseases, as well as the activity of phytochemicals on human proteins. Even though our database contains 20,000 phytochemicals, this is still just a fraction of the natural compound space, which is estimated to be more than 150,000 compounds. Few plants have undergone a complete phytochemical profiling, while the majority has either been studied for specific compounds, if at all. In addition, the biological activity of natural compounds and plants is typically tested experimentally against few, selected proteins or disease phenotypes. Thus, the protein space and the phytochemicals identified in our study as the major players in the colon cancer interaction network, are based on the to date available information in PubMed and may be further revised in the future, as new knowledge on the medicinal properties of plants and their natural compound constituents is going to emerge.

## Conclusion

In conclusion, by developing a systems chemical biology platform that integrates data from the scientific literature as well as online and *in-house* databases we revealed novel associations between dietary molecules with candidate colon cancer targets. Nevertheless, the methodology proposed here for understanding which processes involved in the onset, incidence, progression and severity of colon cancer are modulated by dietary components, is applicable to any large-scale diet-disease association study, where information about the small molecule constituents of the diet is available.

## Methods

### Plant, phytochemical and protein target data

In the study of Jensen *et al.,* (2014) [11] we applied text mining and Naïve Bayes classification to assemble information on plant-phytochemical and plant-disease associations. The 158 plants that were used in this study are the ones showing the highest probability (p = 1) of a positive association with colon cancer. From the same *in-house* database we extracted the chemical composition of each plant (3,526 unique phytochemicals) and after standardization, by removing salts, ions and hydrogen atoms, an InChi key was generated for unique identification.

Proteins forming the candidate colon cancer target space were retrieved from three different sources: (i) from the National Cancer Institute we retrieved all drugs approved by the FDA for treatment of colon cancer (http://www.cancer.gov/cancertopics/druginfo/colorectalcancer). The protein target of each drug was extracted from DrugBank database [22]; (ii) from the KEGG Pathway Database [23] all proteins from the colon cancer pathway (KEGG Pathway id: hsadd05210) were retrieved; (iii) the colon cancer prognostic signature gene set of 87 mRNA transcripts was taken from Oh *et al.,* (2012) [13]. In addition, we included first-degree neighbors of all the proteins falling in (i) to (iii) using STRING 9.1 [24]. In STRING each interaction is assigned a score based on evidence; here we applied a medium confidence threshold (score > 400). To ensure the biological validity of the interactions in the context of colon cancer only proteins for which there was positive evidence of expression in the colon according to the Human Protein Atlas [14] were included. Protein-protein interactions not derived from *Homo sapiens, Rattus norvegicus* and *Mus musculus* were removed.

### Chemical-protein interactions

ChEMBL [25], a database of manually curated small molecule-protein bioactivities, quantified by a measured experimental value, was used for retrieving interactions of phytochemicals with proteins. The bioactivities were filtered according to Kramer *et al.* (2012) [26]. In the present study, only K_i_, IC_50_, potency, inhibition, EC_50_ and K_d_ from experiments performed on proteins from *Homo sapiens, Rattus norvegicus* and *Mus musculus* were included. To accommodate for multiple measurements of the compound on the same protein, we calculated a probability (based on frequency) that the compound had an effect on the protein using the equation below:

A threshold was set as follows for the various kinds of pharmacological measurements: for Ki, EC50, IC50 and Kd, a compound was deemed to interact with the protein if the pChembl value (corresponding to the -log10([M]) value) was greater than 5.5; for inhibition, a compound was deemed to interact with the target if the percentage value was greater than 20; for potency, a compound was deemed to interact with the target if the micro molar value was lower than 500 μM. A single experiment was defined as “positive”, i.e. the compound interacts with the protein, if the measured value was above the threshold. Only compounds for which the positive evidence outweighed the negative evidence (i.e. P ≥ 0.5) were included for further analysis. The ChEMBL database was searched for both exact compounds using the InChI key and similar compounds using a Morgan circular based fingerprint and comparing compounds by the Tanimoto coefficient (Tc). Two compounds were deemed similar if Tc ≥ 0.85 with a difference in molecular weight lower than 50 g/mol and were thus expected to show approximately the same behavior against the same set of proteins.

### Chemical similarity between phytochemicals, drugs and metabolites of the colon metabolic network

The phytochemical space was compared to all approved drugs retrieved from Drugbank [22] and human metabolites involved in reactions in the colon [12]. For every compound, we computed a 1024 bit Morgan circular fingerprint, Molecular Weight (MW), Topological Polar Surface Area (TPSA) [27] and Octanol/Water coefficient (SlogP ) using the KNIME [28] RDKit plugin. Using each descriptor, a matrix of 1027 columns was constructed, in which each row represented a drug, a human metabolite or a phytochemical. Each individual column was scaled to have mean = 0 and standard deviation = 1, to ensure no bias for further distance calculations. We calculated the Euclidian distance between each small molecule, and performed a classical multidimensional scaling (MDS) using the R built-in package cmdscale. Classical MDS is a dimensionality reduction technique, which aims to place objects in a lower dimensional space, keeping the between-object distance as close as possible to the original space. In this case, we choose to represent our 1027 dimensions (molecule features) in a 2 dimensional space.

### Highly targeted protein space and plant efficacy

The pairwise correlation between each pair of proteins was calculated as the ϕ-coefficient. The ϕ-coefficient is a measure between -1 and 1 of correlation between two binary variables, and is related to the χ^2^ distribution as shown below:

where n is the total sample size. p-values were adjusted for multiple testing using the Bonferonni correction. Only correlations with adjusted p-value ≤ 0.05 were considered significant, however biological conclusions that rely on p-values, especially so close to the arbitrary cut-off of significance, should be interpreted with caution and the actual effect size should also be carefully examined before definitive conclusions are made.

For each plant *P*, the efficacy, *E,* of the plant was calculated as:

Furthermore, we calculated a weighted efficacy, *E*_*w*_, which takes into account the number of compounds targeting each protein:

We scaled both the weighted and un-weighted efficacy values between 0 and 1, keeping the relative difference between plants.
